# Designing for flexibility in hybrid care services: lessons learned from a pilot in an internal medicine unit

**DOI:** 10.3389/fmedt.2023.1223002

**Published:** 2023-11-20

**Authors:** Nirit Putievsky Pilosof, Michael Barrett, Eivor Oborn, Galia Barkai, Eyal Zimlichman, Gad Segal

**Affiliations:** ^1^ Cambridge Digital Innovation—CJBS & Hughes Hall, University of Cambridge, Cambridge, United Kingdom; ^2^ Cambridge Judge Business School (CJBS), University of Cambridge, Cambridge, United Kingdom; ^3^ Warwick Business School, The University of Warwick, Coventry, United Kingdom; ^4^ Sheba Beyond Virtual Hospital, Sheba Medical Center, Israel Ministry of Health, Ramat Gan, Israel; ^5^ School of Medicine, Tel-Aviv University, Tel-Aviv, Israel; ^6^ Sheba Medical Center, Israel Ministry of Health, Ramat Gan, Israel; ^7^ Sheba’s Talpiot Medical Leadership Program, Ramat Gan, Israel; ^8^ Education Authority, Sheba Medical Center, Ramat Gan, Israel

**Keywords:** hybrid model, virtual care, telemedicine, healthcare design, internal medicine, hospital-at-home, home hospitalization, digital transformation

## Abstract

Digital transformation in healthcare during the COVID-19 pandemic led to the development of new hybrid models integrating physical and virtual care. The ability to provide remote care by telemedicine technologies and the need to better manage and control hospitals’ occupancy accelerated growth in hospital-at-home programs. The Sheba Medical Center restructured to create Sheba Beyond as the first virtual hospital in Israel. These transformations enabled them to deliver hybrid services in their internal medicine unit by managing inpatient hospital-care with remote home-care based on the patients’ medical condition. The hybrid services evolved to integrate care pathways multiplied by the mode of delivery—physical (in person) or virtual (technology enabled)—and the location of care—at the hospital or the patient home. The study examines this home hospitalization program pilot for internal medicine at Sheba Medical Center (MC). The research is based on qualitative semi-structured interviews with Sheba Beyond management, medical staff from the hospital and the Health Maintenance Organization (HMO), Architects, Information Technology (IT), Telemedicine and Medtech organizations. We investigated the implications of the development of hybrid services for the future design of the physical built-environment and the virtual technological platform. Our findings highlight the importance of designing for flexibility in the development of hybrid care services, while leveraging synergies across the built environment and digital platforms to support future models of care. In addition to exploring the potential for scalability in accelerating the flexibility of the healthcare system, we also highlight current barriers in professional, management, logistic and economic healthcare models.

## 1. Introduction

New hybrid models of care integrating remote technologies have accelerated opportunities to transform how healthcare is provided and where it is delivered. In this paper, we examine hybrid services as those enabled by both the integration of physical (hospital) beds and virtual (home) beds as well as physical (in person) and virtual (via remote technologies) contact. The dramatic growth of digital health during COVID and the development of remote technologies, including patient monitoring, telehealth, and artificial intelligence (AI) based predictive diagnostics, have boosted the shift towards hospital-at-home services that can be scaled for improved service provisioning (1). Studies show the potential for home hospitalization to reduce costs, health care use, and readmissions and improve patient experience, compared with conventional hospital care (2–4), while recognizing that the system should strive to provide the right balance between hospital and home care and between in-person and remote care. The shift of healthcare services to hybrid models between the hospital and the home, enhanced by remote care integrating physical and virtual spaces, introduces a new approach to healthcare flexibility, transforming and going beyond the concept of a hospital to include the wider healthcare ecosystem.

Designing for flexibility is a major theme in healthcare design (5). The constant and rapid change in healthcare resulting from transformations in medicine, technology, and sociology, demands a design strategy for navigating future change. Consequently, flexibility is often a requirement to be built into the hospital's architectural plan to anticipate the growth and changes of the facility and provide a sustainable, whole-life-cycle approach to the hospital operation (6). The terminology of ‘design for flexibility’ refers to strategies that support versatility, modifiability, convertibility, and scalability to future-proof hospital operations over time (7, 8). Architectural design methods for flexibility in the built environment include loose-fit design, modularity, standardization, system separation, interstitial service spaces, and infill systems. The Open Building approach, which advocates design for flexibility, recognizes different life spans of building elements and proposes distributed decision-making processes and strategies to address the resulting complexity (9, 10).

One of the challenges in healthcare design for flexibility is the need to design highly specialized medical functions, which often conflicts with the need to design the hospital facility to accommodate evolvement and change of functions. John Habraken declared that “Hospitals are functionally complex and subject to frequent change over time. As such they may well pose the most difficult design challenge in contemporary architecture” (10). Accordingly, architecture design strategies provide different approaches to the need to design for a specific medical function while planning for its future change (11–13). Yet, retrospective studies on hospital change over time have revealed that not all design strategies for flexibility stand in practice (14). The studies highlight the influence of healthcare policies, organization culture, and funding models, on the development of strategies at an early stage of the design process, and their impact on the flexibility of hospitals to change in the future (11).

Digital technologies are also designed for flexibility. Development and implementation of medical devices and remote technologies are undergoing constant experimentation and testing to support the evolving requirements of the healthcare industry (15). Digital technologies for acute care at home, for example, are a burgeoning field, with industry partners working to create or adapt their technology solutions across telemedicine, remote patient monitoring (RPM), clinical team coordination, and supply chain management technologies to better enable hospital-level care in the home (16). One of the fields in development designed for flexibility of use is comprehensive Electronic Health Records (EHR), evolving from documentation tools to include functions such as order entry, results management, decision support, and embedded clinical connectivity and virtual care tools (16).

Designing for flexibility in healthcare services by developing new hybrid models of care requires a systemic approach to integrate the design for flexibility in the built environment with the design for flexibility in digital technologies. Some argue that future sociotechnical systems around digital technologies should promote a shift from technology-centered engineering that produces objects and machines with immensely codified and rigid practices to flexible autonomy, considering systems as a representation that articulates concepts of structures, functions, contexts and resources (17). As such, studying the transformation of the built environment as a result of remote technologies and analyzing the dependencies between them can promote the needed flexibility of design in hospitals and across the wider health ecosystem.

## 2. Methods

The exploratory study involved qualitative inquiry, aiming to examine how new hybrid care models, integrating physical and virtual environments, are providing new levels of flexibility in healthcare services as synergies are created between digital technologies and the built environment. We explored these developments through our ongoing research from June 2020, focusing on the pilot for internal medicine home hospitalization from July 2021 to December 2022. The qualitative study is based on participant observation and forty formal interviews with the management of Sheba MC and Sheba Beyond (18), medical staff from the hospital and HMO (8), IT directors (2), Telemedicine and Medtech organizations (6), architects (3), and policymakers at the Israeli Ministry of Health (MOH) (3). The observation included a demonstration of the physician's work at the hospital and the design and operation of remote health platforms by telemedicine companies. The leading researcher also participated in the meeting of the hospital management to approve the architectural design of the hybrid internal medicine unit.

The formal semi-structured interviews included topics such as the use of telemedicine technologies, the change in professional practice, and the impact on hospital design. The interview questions addressed the development of the new model of care, the personal experience of the staff, and the challenges and opportunities for future development. The interview questions focused on the specific role of each interviewee. Most of the interviews were held in person in the hospital and some were conducted virtually via Zoom. Each interview took approximately 30–60 min, most being recorded in Hebrew with the consent of the interviewees, transcribed, and professionally translated into English. The researchers also used interview notes and field observations recordings, analysis of architectural plans, hospital webinars, and media coverage. Thematic qualitative data analysis was adopted to identify emerging themes based on principles of naturalistic inquiry and a grounded approach to conceptual development.

## 3. Case study: Sheba beyond internal medicine home hospitalization program

During the COVID-19 crisis, Sheba MC in Israel, with its ARC Innovation Center, accelerated the use of telemedicine technologies for remote inpatient and outpatient care. The hospital developed new models of care treating COVID-19 patients remotely at their home or within the hospital units to prevent cross-infections and reserve personal protective equipment (PPE) (18–20). To extend their remote care services, Sheba MC launched in 2021 the first virtual hospital in Israel named Sheba Beyond. The new hospital arm developed services of virtual clinics, multidisciplinary online rehabilitation, remote chronic disease management, remote expert consultation, and home hospitalization. The hybrid program for internal medicine home hospitalization was based partly on a collaboration between the hospital, the HMOs’ community-based care services, and the support of the Israeli Ministry of Health. The partnership between the hospital and the community services was possible after changing regulations and reimbursement models by the Ministry of Health to promote home hospitalization and telemedicine adoption.

### 3.1. Using remote technologies to provide care between the hospital and home

Sheba Beyond is physically located at Sheba MC in Ramat Gan, central Israel, but it provides healthcare services outside of the hospital campus. The location of Sheba Beyond headquarters within the hospital's main campus enables easy communication and transportation with both personnel and services of Sheba laboratories and imaging institute. Also, the proximity of Sheba Beyond to the emergency department and central hospitalization tower makes it easy to reach patients newly recruited for hospital-at-home services. The service of Sheba Beyond for internal medicine home hospitalization is provided in the geographical area of central Israel, treating patients within a 50 km range by internal medicine specialists from the hospital with nurses at the patient home, using telemedicine technologies for remote monitoring, diagnostics, and communication. The technologies used at home included TytoCare Physical Examination kit, Sensors for Vital Signs monitoring (pulse, heart rate, oxygen saturation and temperature), Electrocardiogram (ECG), portable x-ray machine and the Datos Health platform to manage the care remotely. The physicians at the hospital also used the hospital EHR system and Datos Health platform for communication with patients and family members.

Between July 2021 and the end of December 2022, the program for internal medicine home hospitalization included 452 patients: 256 Female (57%) with an average age of 73.9 years, and 196 Male (43%) with an average age of 72.3 years. The average LOS in the home-hospitalization program was 3.5 days. In the following 30 days since hospitalization, 68 patients were readmitted to the hospital (15%), and 29 patients were readmitted to home-hospitalization (6%).

At the beginning of the program, the patients were recruited in the Emergency Room (ER), before they were hospitalized in the internal units, but as the program evolved, the team decided to recruit patients from the hospital wards as well. The participants in the program were stable patients with infections requiring hospitalization in an internal medicine unit. Most patients had multi-morbidity conditions, including Cardiovascular disease in 256 (56%) patients, Metabolic or Endocrine conditions in 200 (44%), Malignancy or hematologic conditions in 110 (24%), Autoimmune disease in 56 (12%), Neurologic or Psychiatric conditions in 53(12%), and Respiratory disease in 49 (10%). 335 patients were diagnosed with COVID-19 (74%).

The inclusion criteria were acceptance of informed consent by the patients themselves or their legal guardians and diagnoses that are included in the Israeli Ministry of Health list of suitable cases for home hospitalizations, such as pneumonia, urinary tract infections, cellulitis, exacerbation of chronic obstructive pulmonary disease (COPD) and exacerbation/deterioration of congestive heart failure (CHF). The exclusion criteria included hemodynamic instability (systolic blood pressure <100 mmHg; diastolic blood pressure <50 mmHg, rapid and/or slow irregular heart rhythm (tachyarrhythmia/bradyarrhythmia), evidence of respiratory insufficiency (either hypoxemia <92% at room air or hypercarbia/respiratory acidosis), patients suffering from acute confusion (delirium), those with severe electrolyte imbalances in their blood, and all patients deemed unsuitable for home hospitalization based on the clinical judgment of the ER physician and the Sheba Beyond physicians. Patients without a caregiver present 24 h at home were also excluded.

The clinical team included daily visiting nurses, remote case manager nurses, senior physicians, imaging technicians, and Sheba's medical specialists in disciplines of internal medicine (e.g., nephrologists and infectious diseases) and specialists in other clinical realms such as gynecologists. The primary personnel of the home hospitalization service included three specialist physicians in varying partial and full employment, and 8–12 nurses, most dedicated only to hospital-at-home services while some also work in other tele-medical services of Shea Beyond. The home-hospitalization service involved investigations at the patient home, including 330 Chest x-Ray examinations, 360 ECG tests, 400 blood tests, and 51 Urine tests. Interventions included 114 intravenous (IV) entries and 105 cases of oxygen support.

### 3.2. Designing an internal medicine unit with physical and virtual beds

**Figure 1:**
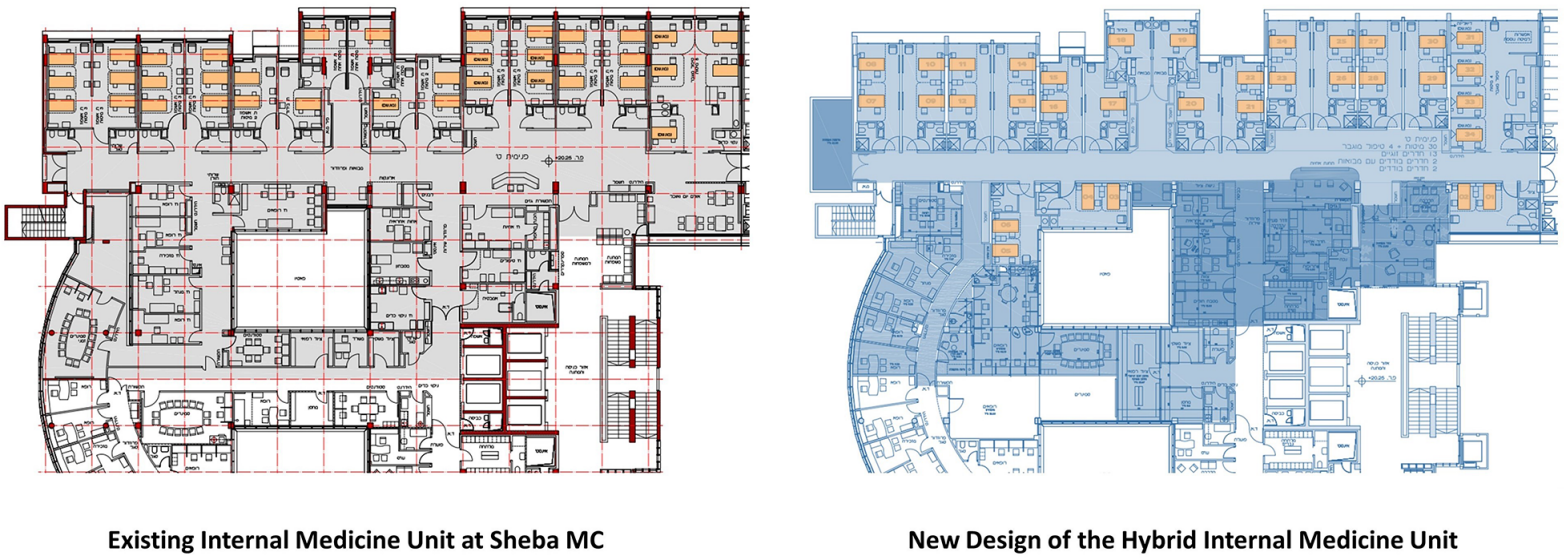
Architectural plans of the existing internal medicine unit at Sheba MC (left), and the proposed renovation plan (right) with fewer physical beds and additional office space for virtual care. Source: Architectural Design by Arch. Tal Einhoren, Chief Architect at Sheba MC, and Faten Kattouf Architects, 2021.

The new service of home-hospitalization led to a conceptual design of a hospital medical unit with physical and virtual beds, integrating inpatient hospital-care with remote home-care. Patients can be admitted in physical beds at the hospital unit or in virtual beds at their home based on their medical conditions and personal preferences. This idea was later developed into a design project aiming to renovate one of the seven internal medicine units in the hospital as a hybrid unit in the future. In a design process that took over a year, a designated team, including medical staff, architects, digital transformation experts, and hospital directors, debated the implications of the model on the medical and nursing protocols, the IT infrastructure, and the design of the physical unit. The results included a specified program, architectural drawings, and 3D illustrations. One of the main changes in the design of the renovated hybrid unit compared to the existing design of internal medicine units in the hospital is the number of patient beds. Although the unit is designed to manage a larger number of patients, the new design of the hybrid unit includes fewer physical beds, made possible by the addition of virtual beds for patients hospitalized at home. The new design incorporates 34 physical beds and 12 virtual beds (a total of 46 beds), compared to 37 physical beds in the existing unit (Figure 1).

Although the renovation was deferred, the design of the new hybrid internal medicine unit illustrates a new approach integrating physical and virtual environments of care. Introducing virtual beds into the design of a hospital unit allows for a reduction in the number of physical beds, providing a higher standard of hospitalization. The new design includes only single or semi-private rooms compared to triple patient rooms in the existing units. Admitting fewer patients per room correlates with evidence from research showing improved outcomes, including an increase in patient safety by reducing the risk of hospital-acquired infections, medical errors, and patient falls, reduced stress, improved sleep, privacy, sense of control, and improved communication with the staff (21–23). The design of the unit also introduces more space for family members and visitors, including an entrance lounge and retreat balconies. For the medical staff, the new design includes more office space including isolated areas for virtual communication of the physicians and interns with their remote patients. The nurse station was designed to support remote communication and monitoring of patients, whether they are located within the unit or at their home.

## 4. Results

The Sheba MC study revealed the transformation process of the hospital to support new models of remote care. Although the transformation is a pilot at a preliminary stage of development, it provides insight into the future potential as well as challenges in moving toward a new era of hybrid care services. The study specifies how the integration of flexibility in the built environment along with flexibility in digital technologies, facilitates new service pathways across the hospital and home.

### 4.1. Flexibility in the built environment

The study illustrates how the hybrid care services enhanced flexibility across the built environment. Inpatient care is no longer dependent on the capacity of the hospital building to accommodate patients but can be expanded to include the homes of the patients in their community settings. Such flexibility allows an increase in the number of patient beds beyond the limitation of the hospital facility by using the patient bed at home. This ability to expand acute care capacity is significant for the healthcare system in Israel, which has a relatively low number of hospital beds per population compared to the OECD countries with an increasing shortage expected in the next decades (24, 25).

“I*t is a given that we will never have the number of beds or physicians we would like to have for internal medicine care. I understood, as have others, that physical beds will not appear in proportion to need, and therefore, remote hospitalization can supply at least a partial solution to that problem*” *(medical director)*.

The flexibility to manage over occupancy showed potential to enhance the resilience of the healthcare system in times of crisis, such as future pandemics or over-occupancy in the internal medicine units in the wintertime.

“*The hybrid model allowed us to manage over-occupancy during the winter surge by hospitalizing patients in their homes. It has improved the patient experience, as it is always better to be in your personal bed at home than to be located in the hospital with another patient in the room or at the unit's corridor*” *(hospital manager)*.

As the hybrid care services are still evolving, the design requires flexibility to support diverse functional programs. For example, the hospital management questioned what should be the future ratio between critical and intermediate care in internal medicine units, and the ratio between physical and virtual beds. When the management considered different options to scale the new hybrid model they wondered if they should add virtual beds to each one of the existing seven internal medicine units or develop a separated virtual unit with only virtual beds for patients at home. This option was deferred because of the internship model of the hospital, posing a challenge to train interns in a virtual unit, and the need to recruit an experienced team.

The design of the internal medicine unit also highlighted the need to provide maximum flexibility in future design of hospital medical units, to support change in the function of rooms between clinical and support spaces, including offices and social areas. It indicated the evolving model of the nurse station into a control center, extending the visibility of patients by telemedicine technologies beyond the limitations of physical oversight. This has implications for the location of patients in the care unit, independent of distance from the nurse station. In addition, recognizing the potential of the hybrid model to move moderately ill patients to their homes, the hospital medical unit will have to accommodate an increase in severely acute patients. These changes in requirements by location will require flexibility in the design of the patient rooms and their infrastructure to support the different levels of care.

### 4.2. Flexibility enabled by remote technologies

In addition to providing flexibility in the built environment, our study identified the concomitant need to design remote technologies to flexibly organize hybrid care services. For example, the health platform was designed as an enterprise system to reconfigure different hybrid care services.

“*We can decide if the system is going to be relevant to different medical services or to a specific one. The system has to be generic and personal at the same time. It is controlled by parameters and feature flags so the users can configure the system to their specific needs*” *(director of a remote technology company)*.

The RPM system also includes a patient care app and a care-team dashboard. Each part has different features based on the program of care.

“*We have the challenge of creating and managing operational programmes for both patients and providers at the same time. Medical directors need to define what is the best way for them to utilise the system for the program, what kind of engagement they need with the patient and among the care team. They often need to rethink their processes and transform their professional behaviour*” *(VP product of a remote technology company)*.

The physical examination technology provides additional system solutions as part of the remote care infrastructure. The core technology was initially designed to allow the patient to perform guided medical exams with a remote healthcare provider. In supporting hybrid care models, they developed their device into two products for patients and medical professionals. The former was designed for family use in home-based consultation, while the latter was developed for home hospitalization, and allowed the nurse visiting the patient at home to assist in remote examination by the physician at the hospital.

“*We design one product for the patient in an appealing and user-friendly way. We needed to give the patient the confidence they can do the examination themselves. The second product, on the other hand, was designed for the physicians as a medical device. The sensors are the same but more durable and look professional to give the physician confidence to provide diagnosis*” *(VP global business development and sales of a digital technology company)*.

The platform of the remote examination technology was designed for flexibility in integrating diverse data sets, including unstructured data like videos, images, and sound, from different monitoring devices.

“*We added capability to integrate other devices of various companies to our platform. Home hospitalization sometimes requires measuring other conditions, like an ECG, and we did not want to limit the monitoring capacities of the hospital and the HMO*” *(remote technology developer)*.

The flexible redesign of care around hospital-at-home demands new skills of participants to have requisite technological abilities in using the system and providing ongoing support. In some cases, the nurses that came to the patient home were responsible for training, showing the family how to use the technologies, and in other cases, technology personnel did the training from a distance.

“*We try to train patients before they are moved from inpatient care to home hospitalization, but it is often not enough. They are so confused, get instructions for their treatment, have to organize at home, and are sick. So, they will often not remember many things, and we have to reinforce it*” *(Medical director)*.

While some technologies provide flexibility in their user interface, there was a significant challenge in the integration of the different remote technologies and data sets. This was further complicated by the lack of integration of the EHRs at the hospital and HMO, each having diverse security and privacy measures. This forced the care team to use multiple computers to integrate the data in navigating and providing care for the patients.

“*Sheba and Maccabi have different digital systems. Prof. S. updates both the hospital and the Maccabi system to record everything he does. He makes a note of all the tests and emphasizes all of the important issues to ensure we are synchronized. This demands double work on his side*” *(nurse from Maccabi)*.

Despite these systems integration challenges, our findings also highlighted the potential affordance of integrating telemedicine technologies with EHRs, and the subsequent use of AI and analytics to predict the patient's medical needs and the disease trajectory in navigating treatment pathways. Further, the future use of telemedicine is envisioned to provide a streamlined, even seamless coordination of patient care while promoting efficiency of operations across the hospital and home.

“*I envision that in the new unit, when you enter the doctor*’*s room and you see his screens, you will not know which patient is tele (hospitalized at home) and which is in room 12 (hospitalized at the hospital). Some criteria might be diverse, but the system does not necessarily have to be different*” *(internal medical director)*.

### 4.3. Flexible models of care through hybrid services

**Figure 2:**
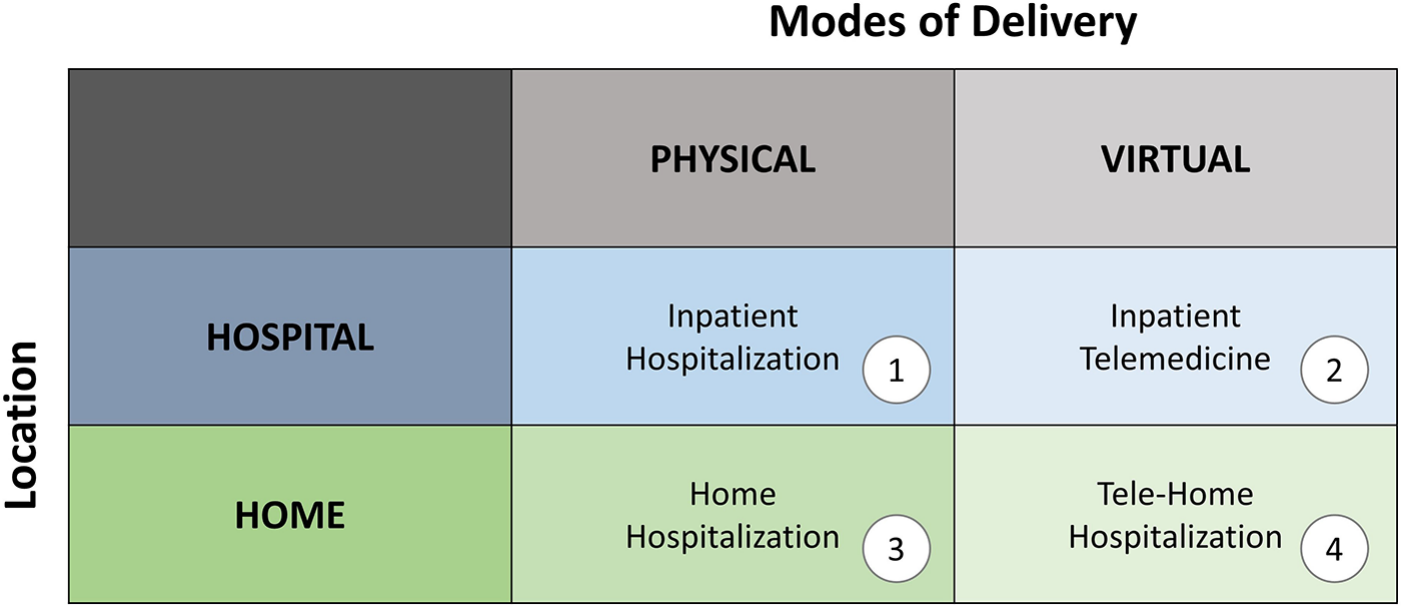
Matrix of care pathways multiplied by the mode of delivery—physical or virtual, and the location of care—at the hospital or the patient's home (the authors).

The study revealed how hybrid care services across physical and virtual environments, enabled by remote technologies, facilitate flexibility in healthcare services. In the new model, the care pathways are multiplied by the mode of delivery, either physical or virtual, and by the location of care, either at the hospital or at the patient's home. This results in four main care pathways: (1) inpatient hospitalization, (2) home hospitalization, (3) inpatient telemedicine, and (4) tele-home hospitalization (Figure 2).

The ability to move between the four care pathways based on the patient's medical condition and personal preferences enhances the flexibility of the healthcare system to offer patient-centered care. Our case focuses on the shifts in care between inpatient hospitalization (mode 1) and tele-home hospitalization (mode 4) via home hospitalization (mode 2), while earlier work has focused on inpatient telemedicine (mode 3) (18–20). An important focus in this transition is on the continuity of care as the same clinical team is responsible for the patient at home and at the hospital in case of deterioration in their medical conditions when the patient is re-admitted. The collaboration between the hospital and the HMO in the community presented the potential to overcome silos in the patient journey, thereby enabling this flexibility in care pathways.

“*Telemedicine and home hospitalization hold the potential to bring about the first real connection between internal medicine and community-based care*” *(medical director)*.

The option for home hospitalization improved patient and family experience by providing privacy and a sense of control at home. The physicians reported a high level of satisfaction from the patients and their family members, confidence in the quality of care they provided, and possible reduction in hospital complications such as infections, delirium, and fall injuries.

“*Virtual care at home is often better for the patient, as it is provided by a medical expert compared to physical care by an intern at the hospital unit*” *(hospital manager)*.

In addition, the new model can also provide more personalized care, allowing the patient to choose the treatment place and the family to be active partners in the care process.

“*The home environment is often very comforting. I feel that in almost every in-home hospitalization visit I attend. I once paid a visit to a man, a young married man with small children, and as I administered the treatment, his two-year-old son was lying next to him in bed. The child brought him toy cars and played with him while he was receiving the IV. That couldn't happen in a hospital. The effect on the patient*’*s mood and recovery is totally different*” *(nurse from Maccabi HMO)*.

The hybrid model can support healthcare systems dealing with a lack of staff by providing flexibility in staffing enhanced by remote consultations and managing changes in patient loads by offering digital tools to supervise and monitor more patients remotely. In home hospitalization, the family often becomes part of the care team, adding workforce to healthcare services.

“*In the design of inpatient units, we usually try to separate the movement of the physician from the movement of the family because we don't want them to interact and cause delays or interruptions. In telemedicine, it is a whole other state of mind because you are interested in interaction with the family, serving as part of the staff*” *(healthcare architect)*.

The new hybrid model also demonstrated flexibility in the organization of the hospital and the HMO to adjust internal processes and protocols and invent new models for collaboration. For example, the hospital shifted standard practices of inpatient hospitalization, in moving patients to home hospitalization after 48 h or during the last days of their admission where care is mostly for supervision and support. This was possible since the hybrid model provided a sense of control and safety, as the patient continued to be monitored and could be readmitted at the hospital unit if needed.

“*We can leap (frog) hospitalization (at the hospital) or choose a model of ‘half-way out’ (moving the patient to home hospitalization after a few days) based on the individual situation at hand*” *(internal medicine physician)*.

“*In internal medicine, the best time to be discharged is after the first 48 h, because the main diagnostic effort is made during the first 48 h. There could still be (clinical) question marks after that, but much less, since all of the essential testing should be done within the first 48 h*” *(internal medical director)*.

Hybrid care services integrating hospital and home hospitalization can also provide flexibility in economic models, based on variation in the length of stay calculation. The option to discharge and readmit patients at home based on the level of care, can save the cost of hospitalization days.

“*The model provided an entirely new approach for me, being able to hospitalize and discharge and re-hospitalize according to the patient condition at home. This is not possible in traditional hospitalization models because of the bureaucracy involved and the complex process of discharging, resulting in redundancy of hospitalization days. At home, hospitalization does not have to be continuous*” *(internal medical director)*.

While the model presented flexibility in healthcare services and potential to optimize operations and enhance the experience of patients, family, and caregivers, the model has not yet been integrated and scaled to all the internal medicine units at the hospital. One of the challenges has been the need to recruit internal medicine physicians to the program.

“*The program reached a proof of concept, but it faced limitations to scale due to opposition on behalf of other medical directors. We also had a problem to recruit physicians in internal medicine, which is considered the ‘Queen of Medicine’… They are trained to treat patients physically and it*’*s hard for them to transform to virtual care*” *(medical director)*.

“*To scale the model, we need to overcome professional resistance and change organization culture. We need an early adapter and a big believer to lead the change*” *(the director of the program)*.

Despite the pilot showing the potential for realizing flexible models of care, the program for internal medicine home hospitalization has not scaled yet, and the renovation of the internal unit has been deferred. Still, the extent and scope of hybrid services is implemented in other medical programs.

“*Currently, the collaboration pilot with the HMO has ended due to reimbursement challenges and the HMOs interest to provide their own home-hospitalization services. However, the model is now being developed and implemented in Pediatrics, Psychiatry, and Women*’*s Care*” *(medical director)*.

Nonetheless, these capabilities of telemedicine and remote patient monitoring developed through Sheba Beyond during the COVID-19 pandemic and advanced in their program for hospital-at-home, were also used widely during the deployment of the national Israeli field hospital in Ukraine in 2022. The field hospital was installed as an additional unit of Sheba MC, connected to the digital platform of the hospital, enabling remote consultation with the most experienced specialists in Israel (26). Extending the hybrid model across two countries demonstrated the added value of flexibility in healthcare services in times of crisis.

## 5. Discussion and conclusions

The study explored an innovative approach to integrating physical and virtual environments in the design of an internal medicine unit using remote technologies as well as in person treatment for hybrid care services. The case illustrated the possible implications of remote care on the transformation of healthcare systems and the design of future hospitals. It showed the potential to advance patient-centered care, personalized care, shift management and economic models of hospitalization. Specifically, it indicated a new approach to design for flexibility in healthcare services. The study revealed how flexibility in the built environment with flexibility in digital technologies facilitated new service pathways across the hospital and home. The extended flexibility in hybrid care services, tested during the COVID pandemic and winter time with high demands, showed potential to enhance the resilience of the healthcare system in times of crisis. This is significant in an era when healthcare systems are considering possible strategies to cope with increased demands, lack of healthcare professionals (27), rising costs of care, and outdated medical facilities. Still, more research is needed to examine the quality of care, patient and staff safety, and the experience of all the stakeholders.

Planning for change and designing for flexibility has always been a major challenge in healthcare design (5–14). Yet, in most cases, the requirement was conceived as being only for the architecture of the building to support change over time. This case demonstrates how digital technologies increase the flexibility of the hospital beyond its physical space into virtual environments, expanding the boundaries of the hospital building into multiple settings across the built environment. The case also brought to light synergies in the design for flexibility of the built environment and design for flexibility in remote technologies, and the importance of managing interdependencies between them. Together, they can stimulate and flexibly manage change in capacity, variation in the functional program, dynamics in operations, and shift in practices while enhancing user experience. In so doing, they provide flexibility in the design of care services to better support patients, family members, staff, and healthcare organizations.

The flexibility to choose between multiple care pathways—physical or virtual care and the location of care at the hospital or home—can support personalized patient care throughout the various phases of the patient journey, including diagnosis, treatment, and rehabilitation. This choice of care provision, whether determined by the medical staff or the patients and their families, can enhance the quality of care, healing processes, human experience, and the efficiency of organizations. However, these emergent and multiplicative sets of options add to the complexity of the healthcare system, leaving the caregivers, the patients, and their families with a need to choose the best care pathway and to mitigate tradeoffs between conflicting interests. An important implication of our research is that stable internal medicine patients can be managed at home if they have adequate family/carer support. Yet, the study also highlighted how physician competence with using various digital technologies for safe and accurate remote treatment is an important factor in being able to develop virtual bed forms of care.

Future research could usefully examine the management and control of the complexity of operations across multiple pathways of care, as hospitals will need control systems to navigate between physical and virtual environments. The case illustrated implications for the design of the built environment, including the need for more office space and redesign of the nurse station as a control center to manage physical and virtual care. Similar to the diverse design models of nurse stations—centralized vs. decentralized (28–30)—control centers can also be designed per unit, or centralized per medical division or hospital building. In all scales and levels, control systems can benefit from using digital technologies such as Digital Twins to improve operational control and patient experience (31). Further, integrating the data of users, services, and environments—physical and virtual—can improve efficiency through real-time analytics and prediction models and support the design of evolving future hybrid care services between the hospital and home.

## 6. Limitations

A boundary condition of our research is that it does not provide the perspective of patients and family members. Future studies should examine the experience of all the involved stakeholders. Also, more studies on design for flexibility in hybrid care services and the implementation of telemedicine technologies in scale and in different programs are required, as well as various medical specialities and levels of care. Further research on the impact of hybrid models of care on the design of healthcare facilities and digital technology in diverse environmental, cultural, and economic contexts will enhance the knowledge base needed for design for flexibility in future development of healthcare architecture and digital technologies for remote care.

## Data Availability

The raw data supporting the conclusions of this article will be made available by the authors, without undue reservation.
